# Supplementary material for: Sociodemographic and Work-Related Variables Affecting Knowledge of, Attitudes toward, and Skills in EBNP of Nurses According to an EBPPQ
Source: Int J Environ Res Public Health. 2022 Jul 13;19(14):8548. doi: 10.3390/ijerph19148548 (PMC9320299; doi:10.3390/ijerph19148548)
Supplement: Supplementary file 1 [file ijerph-19-08548-s001.zip › ijerph-1767631-supplementary.pdf]

International Journal of Environmental Research and Public Health

MDPI

Table S1. Education- and work-related profile of the study group (N=830)

**Variable**		**n**	**%**
Type of higher education institution	Higher vocational school	140	16.9
	Medical university	400	48.2
	Non-medical university	154	18.6
	No data available	136	16.4
Specialisation training completed	No	133	16
	Yes	697	84
Post-graduate training organisation	Private organisation	338	40.7
	Nurse chamber	156	18.8
	Higher education institution	28	3.4
Employment relationship	Contract of employment	703	84.7
	Individual civil law agreement	100	12
	Collective civil law agreement	4	0.5
	No data available	23	2.8
Work system	One shift	298	35.9
	Two shifts	508	61.2
	No data available	24	2.9
Number of jobs	1	474	57.1
	2	248	29.9
	3 and more	63	7.6
	No answer	20	2.4
	No data available	25	3
Place of work	University hospital	213	25.7
	Voivodship hospital	188	22.7
	Municipal hospital	108	13
	County hospital	172	20.7
	Nursing home	11	1.3
	Primary healthcare facility	140	16.9
	Curative care centre	29	3.5
Ward	Paediatric	129	15.5
	For medical treatment	276	33.3
	For non-invasive treatment	165	19.9
	Intensive care unit, emergency unit	119	14.3
	Other	4	0.5
	No data available	137	16.5
Work organisation system	Functional care model	597	71.9
	Small-team model	49	5.9
	Primary nursing	18	2.2
	Other	91	11.0
	No data available	75	9
Work experience [years]	Up to 1 year	32	3.9
	1–10	209	25.2
	11–20	154	18.6
	21–30	270	32.5
	>30	130	15.6
	No data available	35	4.2

Table S2. Regression analysis for the full multivariate model for the subscale 'Level of knowledge of scientific research terminology'

**Variable**	**Level**	**β**	**+95%CI**	**-95%CI**	**t**	***p*-value**
Intercept					5.714	<0.001
Age		-0.16	-0.40	0.08	-1.299	0.195
Work experience		0.08	-0.16	0.33	0.682	0.496
Trainings/courses	No (ref.)					
	Yes	0.00	-0.09	0.10	0.097	0.922
Specialization	No (ref.)					
	Yes	0.04	-0.06	0.14	0.777	0.438
Trainings organized by:	University (ref.)					
	Private organization	-0.03	-0.13	0.07	-0.581	0.562
	Board of Nursing	-0.08	-0.18	0.02	-1.494	0.136
Employment relationship	Civil contract (ref.)					
	Employment contract	-0.10	-0.20	0.00	-1.918	0.056
Number of workplaces	1 (ref.)					
	>1	-0.02	-0.13	0.08	-0.465	0.643
Workplace	Clinical hospital	-0.01	-0.18	0.16	-0.104	0.917
	Provincial hospital	-0.00	-0.16	0.16	-0.011	0.991
	City hospital	0.06	-0.08	0.19	0.834	0.405
	District hospital	0.01	-0.15	0.16	0.065	0.948
	Residential home	-0.00	-0.10	0.10	-0.076	0.939
	Primary care center	0.00	-0.12	0.12	0.016	0.987
	Residential medical care facility	-0.03	-0.13	0.06	-0.656	0.513
Hospital ward	Other (ref.)					
	Paediatric	-0.08	-0.26	0.10	-0.899	0.369
	Surgical	0.02	-0.18	0.22	0.230	0.818
	Non-invasive treatment	0.05	-0.13	0.23	0.578	0.564
	Intensive Care Unit (ICU), Emergency Department	-0.05	-0.22	0.11	-0.656	0.512
Work organization system	Other (ref.)					
	Functional Model of Care	-0.07	-0.22	0.07	-0.984	0.326
	Small-Team Model	-0.08	-0.22	0.06	-1.184	0.237
	Primary Nursing	0.08	-0.10	0.26	0.871	0.384
Is EBNP used in the workplace?	No (ref.)					
	Yes	0.02	-0.10	0.14	0.277	0.782
Did she/he had the subject of EBNP in the course of education?	No (ref.)					
	Yes	0.18	0.07	0.29	3.282	0.001
Does she/he use EBNP in their work?	No (ref.)					
	Yes	0.24	0.11	0.36	3.773	<0.001

β - standardized regression coefficient, CI - confidence interval, ref. - reference level

Table S3. Regression analysis for the stepwise model for the subscale 'Level of knowledge of scientific research terminology'

**Variable**	**Level**	**β**	**+95%CI**	**-95%CI**	**t**	***p*-value**
Intercept					33.701	<0.001
Employment relationship	Civil contract (ref.)					
	Employment contract	-0.10	-0.19	-0.01	-2.129	0.034

Did she/he had the subject of EBNP in the course of education?	No (ref.)					
	Yes	0.21	0.11	0.32	4.088	<0.001
Does she/he use EBNP in their work?	No (ref.)					
	Yes	0.23	0.13	0.33	4.424	<0.001

β - standardized regression coefficient, CI - confidence interval, ref. - reference level

Table S4. Regression analysis for the full multivariate model for the subscale 'Frequency of using various EBNP components in everyday clinical work'

**Variable**	**Level**	**β**	**+95%CI**	**-95%CI**	**t**	***p*-value**
Intercept					6.178	<0.001
Age		-0.23	-0.48	0.02	-1.802	0.072
Work experience		0.07	-0.19	0.32	0.519	0.604
Trainings/courses	No (ref.)					
	Yes	0.01	-0.09	0.12	0.242	0.809
Specialization	No (ref.)					
	Yes	0.02	-0.09	0.13	0.397	0.692
Trainings organized by:	University (ref.)					
	Private organization	-0.02	-0.12	0.09	-0.306	0.760
	Board of Nursing	-0.05	-0.15	0.06	-0.861	0.390
Employment relationship	Civil contract (ref.)					
	Employment contract	-0.06	-0.17	0.04	-1.147	0.252
Number of workplaces	1 (ref.)					
	>1	0.07	-0.04	0.17	1.189	0.235
Workplace	Clinical hospital	0.05	-0.12	0.23	0.589	0.556
	Provincial hospital	-0.03	-0.19	0.14	-0.309	0.757
	City hospital	-0.01	-0.15	0.13	-0.166	0.868
	District hospital	-0.06	-0.22	0.09	-0.803	0.423
	Residential home	-0.01	-0.12	0.09	-0.251	0.802
	primary care centers	-0.01	-0.14	0.11	-0.180	0.857
	Residential medical care facility	0.05	-0.05	0.15	1.019	0.309
Hospital ward	Other (ref.)					
	Paediatric	0.03	-0.16	0.21	0.281	0.779
	Surgical	0.08	-0.13	0.29	0.738	0.461
	Non-invasive treatment	0.08	-0.11	0.27	0.872	0.384
	Intensive Care Unit (ICU), Emergency Department	-0.02	-0.19	0.15	-0.210	0.834
Work organization system	Other (ref.)					
	Functional Model of Care	-0.01	-0.16	0.14	-0.106	0.916
	Small-Team Model	-0.01	-0.15	0.14	-0.087	0.931
	Primary Nursing	-0.06	-0.24	0.13	-0.615	0.539
Is EBNP used in the workplace?	No (ref.)					
	Yes	0.04	-0.08	0.17	0.665	0.507
Did she/he had the subject of EBNP in the course of education?	No (ref.)					
	Yes	0.03	-0.08	0.14	0.521	0.603
Does she/he use EBNP in their work?	No (ref.)					
	Yes	0.17	0.04	0.30	2.627	0.009

β - standardized regression coefficient, CI - confidence interval, ref. - reference level

Table S5. Regression analysis for the stepwise model for the subscale ''Frequency of using various EBNP components in everyday clinical work'

Variable	Level	β	+95%CI	-95%CI	t	*p*-value
Intercept					14.936	<0.001
Does she/he use EBNP in their work?	No (ref.)					
	Yes	0.20	0.10	0.30	4.069	<0.001
Age		-0.17	-0.27	-0.07	-3.457	0.001

β - standardized regression coefficient, CI - confidence interval, ref. - reference level

Table S6. Regression analysis for the full multivariate model for the subscale 'EBNP-related skills'

Variable	Level	β	+95%CI	-95%CI	t	*p*-value
Intercept					6.968	<0.001
Age		-0.18	-0.43	0.07	-1.379	0.169
Work experience		0.15	-0.10	0.40	1.182	0.238
Trainings/courses	No (ref.)					
	Yes	0.05	-0.05	0.15	0.932	0.352
Specialization	No (ref.)					
	Yes	0.04	-0.06	0.15	0.800	0.424
Trainings organized by:	University (ref.)					
	Private organization	0.01	-0.10	0.11	0.151	0.880
	Board of Nursing	-0.14	-0.24	-0.03	-2.594	0.010
Employment relationship	Civil contract (ref.)					
	Employment contract	-0.07	-0.18	0.03	-1.397	0.163
Number of workplaces	1 (ref.)					
	>1	-0.14	-0.25	-0.03	-2.506	0.013
Workplace	Clinical hospital	0.02	-0.16	0.19	0.211	0.833
	Provincial hospital	-0.04	-0.21	0.13	-0.460	0.646
	City hospital	-0.01	-0.15	0.13	-0.115	0.908
	District hospital	0.03	-0.13	0.18	0.343	0.731
	Residential home	0.07	-0.03	0.17	1.324	0.186
	primary care centers	0.00	-0.12	0.13	0.071	0.944
	Residential medical care facility	0.02	-0.08	0.13	0.483	0.629
Hospital ward	Other (ref.)					
	Paediatric	-0.08	-0.26	0.11	-0.807	0.420
	Surgical	-0.10	-0.30	0.11	-0.921	0.358
	Non-invasive treatment	-0.15	-0.34	0.04	-1.554	0.121
	Intensive Care Unit (ICU), Emergency Department	-0.06	-0.23	0.11	-0.719	0.473
Work organization system	Other (ref.)					
	Functional Model of Care	-0.10	-0.25	0.05	-1.279	0.202
	Small-Team Model	-0.01	-0.15	0.14	-0.131	0.896
	Primary Nursing	0.09	-0.09	0.28	0.991	0.323
Is EBNP used in the workplace?	No (ref.)					
	Yes	0.03	-0.10	0.15	0.441	0.660
Did she/he had the subject of EBNP in the course of education?	No (ref.)					
	Yes	0.09	-0.02	0.20	1.655	0.099
	No (ref.)					

Does she/he use EBNP in their work?	Yes	0.17	0.04	0.30	2.612	0.009

β - standardized regression coefficient, CI - confidence interval, ref. - reference level

Table S7. Regression analysis for the stepwise model for the subscale 'EBNP-related skills'

**Variable**	**Level**	**β**	**+95%CI**	**-95%CI**	**t**	***p*-value**
Intercept					39.879	<0.001
Does she/he use EBNP in their work?	No (ref.)					
	Yes	0.24	0.14	0.33	4.823	<0.001
Number of workplaces	1 (ref.)					
	>1	-0.10	-0.20	-0.00	-2.056	0.040
Trainings organized by:	University (ref.)					
	Private organization	0.02	-0.08	0.12	0.389	0.697
	Board of Nursing	-0.12	-0.22	-0.02	-2.378	0.018

β - standardized regression coefficient, CI - confidence interval, ref. - reference level
